# The Impact of Family Socioeconomic Status (SES) on Adolescents’ Learning Conformity: The Mediating Effect of Self-Esteem

**DOI:** 10.3390/children11050540

**Published:** 2024-04-30

**Authors:** Houyan Li, Bin Xiao, Guandong Song

**Affiliations:** School of Humanities and Law, Northeastern University, Shenyang 110169, China; 2110009@stu.neu.edu.cn

**Keywords:** family socioeconomic status (SES), learning conformity, self-esteem, mediating effect

## Abstract

Objective: This study aimed to investigate the relationship between family socioeconomic status (SES) and adolescents’ learning conformity and self-esteem among a sample of 15–18 year-old high school students. Methods: A survey was conducted on 339 adolescents using measures of family SES, self-esteem, and learning conformity. An intermediary effect model was constructed to examine the mediating mechanism of self-esteem in the impact of family SES on adolescents’ learning conformity. Results: In our study, we observed that male adolescents were more likely to come from families with higher socioeconomic status (SES) and exhibited relatively higher levels of self-esteem compared to female adolescents. However, this finding should be regarded as an observational outcome specific to our study sample and does not directly indicate a causal relationship between gender and family SES or self-esteem. Adolescents from rural areas were more likely to exhibit tendencies towards learning abidance and compliance. Family SES positively predicted self-esteem. The intermediary model indicated that family SES significantly positively influenced learning abidance and compliance, with self-esteem partially mediating the effects at 33.49% and 33.33%, respectively. Family SES negatively affected learning obedience, with self-esteem partially mediating the effect at 39.77%. Conclusion: Among the 15–18 year-old high school student population, family SES not only directly affects learning abidance, compliance, and obedience but also generates an indirect mediating effect through self-esteem.

## 1. Introduction

At the intersection of higher education and social psychology, research on adolescent learning conformity holds a pivotal position. From the perspective of information processing psychology, individual behaviors are regarded as responses to social stimuli or cues. Within this framework, learning is understood as a motivated behavior of adolescents influenced by social information [1], termed as learning conformity [2]. Confronted with diverse motivations for learning among adolescents, scholars have classified them into three types: learning conformity, compliance, and obedience [3]. However, despite the diversity of learning conformity types, discussions on their underlying causes remain relatively scarce. While some studies have touched upon the potential influence of familySES on adolescent learning motivations [4], how to positively guide students from different family backgrounds to stimulate their positive learning motivations, thereby enhancing their subjective initiative and academic achievement [5], remains a topic worthy of further exploration.

Under the influence of social capital theory, the relationship between family background and educational attainment has become a focal point for scholars. Most studies suggest that family SES, as an external support system [6], often indirectly influences adolescent learning behaviors and motivations [7]. However, compared to family SES, self-esteem has a more direct correlation with adolescent learning behaviors and educational outcomes [8]. In recent years, with the integration of research perspectives, scholars have begun to focus on how family SES shapes individual behaviors through its influence on students’ self-esteem [9]. Although some studies have preliminarily explored the effects of family SES and self-esteem on adolescent learning motivations or engagement, few studies have incorporated different types of learning conformity behaviors into the research framework, which undoubtedly weakens the completeness and comprehensiveness of learning motivation research.

In summary, although current research has touched upon the influence of family SES on learning motivations, discussions on learning conformity remain insufficient. Particularly, there is a lack of exploration on how family SES influences learning conformity under different motivational backgrounds, as well as the mediating mechanism of self-esteem in this process. Therefore, this paper aims to address two core questions: first, how family SES influences different types of learning conformity; second, the mediating role of self-esteem in the relationship between family SES and different types of learning conformity. Through in-depth analysis of these issues, we hope to inject new vitality into learning motivation research and provide more comprehensive theoretical support for educational practices.

## 2. Literature Review and Research Hypotheses

(I) Definition and Theoretical Implications of Learning Conformity

Conformity, as a social psychological phenomenon [10], has long been regarded as a manifestation of group behavior [11] or the “herd effect” [12]. Traditional studies on conformity mainly approach it from the perspective of pressure [13], interpreting conformity as consistent behavioral choices individuals make under social pressure. However, with the emergence of information processing theory and humanistic psychology, modern research has begun to imbue conformity with new theoretical implications [14]. Within this framework, conformity is no longer merely a product of pressure but is seen as a motivated choice individuals make after internalizing social stimuli. Individual conformity motives are thus divided into cognitive, affective, and utilitarian types, corresponding to internal and external social stimuli categories.

Building upon this foundation, learning conformity, as a derivative concept, specifically refers to the learning motivation behaviors adolescents exhibit under the influence of various social stimuli during the learning process [3]. Typological studies on learning conformity have gradually become a focal point for scholars. Sun et al., from an external attribution perspective, divide conformity into normative conformity and informational conformity [15]; while Song Guandong et al., from an internal attribution viewpoint, categorize it into rational conformity and irrational conformity [16]. In the latest research advancements, learning conformity has been further subdivided into three types: learning abidance, learning compliance, and learning obedience [3]. Learning abidance reflects adolescents’ intrinsic learning interest, demonstrating the internal consistency between learning willingness and behavior, which embodies cognitive motivational factors. Learning compliance refers to adolescents’ learning to avoid punishment or the influence of authority, indicating a deviation between learning willingness and behavior, which manifests utilitarian motivational factors. Learning obedience involves adolescents learning to please family members or friends, similarly showing inconsistency between learning willingness and behavior, which showcases emotional motivational factors.

Additionally, scholars have begun to pay attention to the potential influence of different social stimuli on learning conformity. Factors such as self-awareness [15], teacher attention [17], peer influence [17], regional differences [17], and gender disparities [18] have been included in the research scope to explore how they intertwine with the three types of learning conformity and jointly influence adolescents’ learning motivation and behavioral choices. Through in-depth research into these factors, we can gain a more comprehensive understanding of the formation mechanism and influencing factors of learning conformity, providing more targeted theoretical guidance for educational practices.

(II) Exploring the Potential Impact of Family SES on Learning Conformity

Family SES, as a significant indicator of a family’s position in the distribution of social resources, encompasses various dimensions, including parents’ education level, household income, occupational prestige, and social networks [19]. These factors collectively constitute a complex and multidimensional microsocial environment that profoundly influences adolescents’ learning motivation and behavioral patterns [20].

In recent years, an increasing number of empirical studies have begun to focus on the relationship between family SES and adolescent learning motivation. These studies generally acknowledge that family SES is one of the important factors influencing adolescents’ learning motivation [21]. Specifically, adolescents from higher socioeconomic status families often have access to more educational resources, receive greater parental support, and therefore exhibit stronger learning willingness and motivation [19]. Conversely, adolescents from lower socioeconomic status families may face challenges in motivation due to limited educational resources, economic pressure, and lower parental education expectations [22,23].

Furthermore, learning conformity behavior, as an important manifestation of learning motivation, has been confirmed to be closely related to family SES. Learning conformity behavior refers to the learning-related motivated behaviors adolescents exhibit under the influence of social stimuli during the learning process. Based on different sources of motivation and behavioral manifestations [3], learning conformity behavior can be divided into three types: learning abidance, learning compliance, and learning obedience [24]. These different types of learning conformity behavior exhibit differences in psychological mechanisms and behavioral characteristics, and therefore may be influenced differently by family SES.

Specifically, family SES may influence different types of learning conformity behavior through the following pathways: Firstly, family’s investment in education and resource allocation may directly affect adolescents’ learning conditions and learning environments, thereby influencing the development of their learning abidance behavior. Secondly, parental expectations and support may play an important role in adolescents’ learning compliance behavior; high expectations and strong support may encourage adolescents to study harder to meet their parents’ expectations. Finally, the emotional atmosphere and parent-child relationship within the family may affect adolescents’ learning obedience behavior; a positive emotional atmosphere and a good parent-child relationship may stimulate adolescents’ intrinsic learning motivation, prompting them to actively engage in learning.

In summary, this paper proposes the following research hypotheses:

**Hypothesis** **1.** *Family SES significantly influences learning conformity across different types of learning conformity behavior.*

By thoroughly exploring the relationship between family SES and different types of learning conformity behavior, as well as their underlying mechanisms, we can gain a more comprehensive understanding of the important role of family factors in the formation and development of adolescent learning motivation, providing more targeted guidance and recommendations for educational practice [21]. Additionally, this offers new perspectives and insights for future research, contributing to the advancement of this field.

(III) Family SES, Self-esteem, and Learning Conformity: Constructing a Mediation Model

Self-esteem, as an individual’s subjective evaluation and emotional experience of self-worth and self-competence, has long been a focus in the field of psychology [25]. Research indicates that self-esteem formation is closely related to the family environment, particularly with family SES playing a crucial role in its development [26]. Individuals with high self-esteem often demonstrate greater resilience and social adaptability in the face of adversity, a viewpoint validated in studies focusing on low-income family backgrounds [27,28].

Moreover, the relationship between self-esteem and learning motivation has been a hotspot of academic research since the 1980s. Numerous studies have explored the influence of self-esteem on individuals’ intrinsic motivation [29], behavioral performance, and psychological resilience. Students with high self-esteem typically exhibit higher academic achievement and learning abilities [30], possessing clearer learning motivations and goal orientation. This process involves psychological mechanisms such as self-regulation [31], self-persuasion, and self-construction, reflecting individuals’ cognitive processing of self-esteem information.

In recent years, empirical research has also begun to focus on the mediating role of self-esteem in the relationship between family SES and adolescent behavior [32]. These studies suggest that self-esteem may play a crucial mediating effect in the process of family SES influencing learning conformity. Building upon this theoretical background, this study posits that self-esteem serves as a critical bridge connecting family SES and learning conformity.

Therefore, this paper proposes the following research hypothesis:

**Hypothesis** **2.** *Self-esteem mediates the effect of family SES on learning conformity.*

By constructing a mediation model incorporating family SES, self-esteem, and learning conformity, we can gain a deeper understanding of how family factors influence adolescent learning conformity behavior through their impact on self-esteem levels. The construction of this model not only enriches and advances learning motivation theories but also provides valuable guidance and recommendations for educational practice.

## 3. Research Design

(I) Research Subjects

This study centers on high school students from Shenyang Second High School, Shenyang Fifth Senior High School, which are both key high schools, and Shenyang Art Experimental High School, a regular high school. The age group specifically targeted is 15–18 years old. This setting lays the groundwork for subsequent comparisons between key and regular high schools by providing necessary data. To ensure comprehensive and in-depth research, a blend of purposive and stratified sampling methods was utilized, aiming to encompass a wide array of academic disciplines, including liberal arts, science, and the arts.

The questionnaire dissemination process commenced towards the end of April 2023 and culminated at the close of May, encompassing a one-month period. During this timeframe, a total of 355 questionnaires were distributed and subsequently retrieved. Following rigorous screening and validation, which involved the elimination of incomplete or logically inconsistent responses, a final cohort of 339 valid questionnaires was obtained. This reflects a remarkable response rate of 95.5%, indicating a high level of engagement and participation from the targeted 15–18-year-old high school student population.

In terms of sample composition, we have strived for a balanced distribution across gender, grade, and academic discipline. Specifically, male students constitute 54.74% of the sample, while female students comprise 45.26%. Regarding grade distribution, freshmen account for 34.74%, sophomores represent 33.16%, and seniors make up 32.10% of the cohort. In terms of academic disciplines, liberal arts students constitute 24.47%, science students comprise 49.74%, and those pursuing artistic fields represent 25.79% of the sample. This distribution not only ensures the stability and reliability of the data but also provides a solid foundation for subsequent data analysis.

Furthermore, a comprehensive analysis of the students’ familial backgrounds was conducted. The results indicate a wide range of parental education levels, encompassing various educational tiers. This diversity further validates the representativeness and adequacy of our data selection.

In conclusion, through carefully crafted questionnaires and scientific sampling techniques, this study has successfully gathered a high-quality dataset. We are confident that these data will provide valuable insights into the learning landscapes of high school students and the factors that influence their academic performance.

(II) Research Instruments

The research instruments employed in this study encompass four primary components:

Personal Information Sheet: This section gathers essential demographic data from participants, including gender, academic year, place of origin, field of study, as well as parental educational backgrounds and family income.

Family SES Questionnaire: Inspired by Ren Chunrong’s classification approach [33], this survey instrument evaluates a family’s SES through criteria including parental academic backgrounds and household earnings. The educational attainment of parents is segmented into seven distinct levels [34], mirroring the prevalent educational structure, progressing from “no formal education” to “postgraduate” studies. Each level corresponds to a numerical value spanning from 1 to 7. For assessing household income, we employ a five-tier scale, clearly delineated as “low”, “medium”, and “high” income brackets, with numerical values assigned from 1 to 5.

Self-esteem Scale: Utilizing the Chinese adaptation of Rosenberg’s self-esteem scale [35], this section comprises ten statements rated on a Likert scale ranging from 1 (strongly disagree) to 4 (strongly agree). Scores are totaled, yielding a range of 10 to 40, indicative of an individual’s self-esteem level. Rigorous psychometric evaluation affirms the scale’s reliability (Cronbach’s alpha = 0.982), validity (Kaiser-Meyer-Olkin = 0.978; Bartlett’s test *p* < 0.001), and appropriateness for this study.

Learning Conformity Scale: Innovatively crafted based on Song et al.’s conformity taxonomy and Sun et al.’s behavioral conformity framework [3], this scale captures nuances of learning conformity behavior. Employing a 5-point Likert scale, items encompass learning abidance (7 items), learning obedience (6 items), and learning compliance (5 items). Example statements include “My enthusiasm for learning comes from within” (learning abidance), “I strive academically to earn rewards like scholarships” (learning obedience), and “Academic success brings honor to my family” (learning compliance). Robust reliability metrics (Cronbach’s alpha: abidance = 0.965, obedience = 0.973, compliance = 0.936) and discriminant validity underscore the scale’s fidelity and effectiveness.

In summary, the research instruments deployed exhibit robust psychometric properties, ensuring reliable and valid data collection for this scholarly endeavor [Table 1 and Table 2].

(III) Data Analysis Techniques

For this study, data will be analyzed utilizing SPSS 23.0 software. The analysis will encompass multiple methods including tests for common method bias, descriptive statistical analysis, path analysis, and mediation analysis. The common method bias test will be employed to assess potential biases in self-reported data from the same respondents. Descriptive statistics will provide a comprehensive understanding of the basic characteristics of the sample data. Path analysis will be utilized to examine the relationships among family SES, self-esteem, and learning conformity. Mediation analysis, on the other hand, will help validate the mediating role of self-esteem in the influence of family SES on learning conformity. Through the comprehensive application of these data analysis methods, we aim to delve deeply into the research questions and derive scientifically reliable conclusions.

## 4. Research Results and Analysis

(I) Common Method Bias Test

To ensure the accuracy and reliability of the questionnaire data, we employed the Harman test method to examine potential common method biases. The results revealed that all factor eigenvalues were greater than 1, and after factor rotation, the cumulative variance explained by the first common factor was 37.558%, which did not exceed the standard threshold of 40%. This indicates that there is no significant common method bias issue in the data used in this study.

(II) Correlation Analysis of Variables

In this study, an analysis of the mean, standard deviation, and Pearson correlation was conducted for each variable, as shown in Table 3. The results reveal a significant positive correlation between family SES and both learning abidance and learning obedience, indicating that students from higher socioeconomic status families are more likely to demonstrate stronger willingness to conform and obey in their learning endeavors. Interestingly, a negative correlation was observed between family SES and learning compliance, suggesting that some students may be influenced by other factors during their learning process, leading to a reluctance to engage in compliance-oriented learning behaviors.

Furthermore, a significant positive correlation was found between self-esteem and both learning abidance and learning obedience, indicating that students with higher levels of self-esteem are more likely to exhibit positive attitudes and behaviors toward learning. However, the negative correlation between self-esteem and learning compliance suggests that students with higher self-esteem may tend to maintain independence and resist external learning pressures.

These findings further support the mediating role of self-esteem in the process of learning conformity, wherein differences in self-esteem levels may influence students’ attitudes and behavioral choices toward learning tasks.

Additionally, it was observed that gender has a significant negative correlation with family SES and self-esteem, indicating that females and students from lower socioeconomic status families tend to exhibit more fragile self-esteem. However, there was no significant correlation between gender and learning abidance, learning obedience, or learning compliance.

Moreover, students from rural areas were found to be more inclined towards demonstrating motivation for learning abidance and learning obedience, while students attending key high schools exhibited higher family SES and self-esteem compared to students from regular high schools.

These results provide valuable insights and implications for understanding the complex relationships among family factors, individual traits, and learning behaviors.

(III) Structural Equation Model Examination

(1) Path Analysis

**Figure 1:**
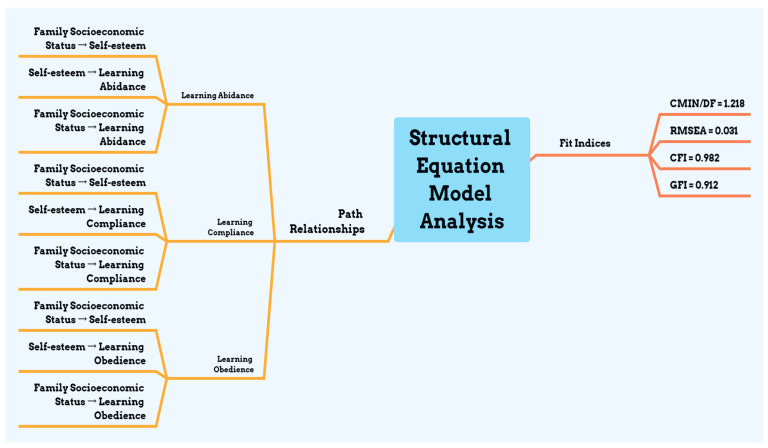
Pathway Analysis in the Structural Equation Model.

In this study, the paths within the structural equation model were analyzed, revealing satisfactory fit indices for the model, specifically with a CMIN/DF of 1.318, RMSEA of 0.033, CFI of 0.986, and GFI of 0.922. Moreover, all paths in the model reached statistical significance standards. The specific path relationships among variables in the research model are illustrated in Figure 1.

Firstly, regarding the path of learning abidance: the influence of family SES on self-esteem was significantly positive (β = 0.516, *p* < 0.01), and self-esteem had a significant positive impact on learning abidance (β = 0.365, *p* < 0.01). Family SES also had a significant positive impact on learning abidance (β = 0.288, *p* < 0.01).

Secondly, concerning the path of learning compliance: self-esteem had a significant positive impact on learning compliance (β = 0.436, *p* < 0.01), and family SES had a significant positive impact on learning compliance (β = 0.709, *p* < 0.01).

**Figure 2:**
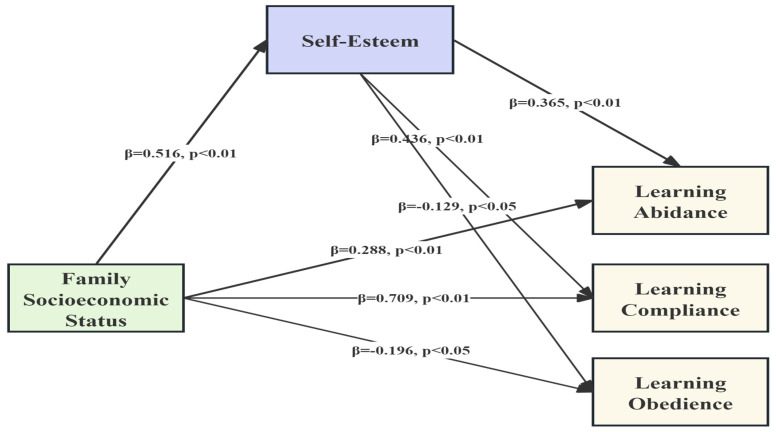
Impact of Family SES and Self-esteem on Learning Behaviors Path Analysis.

Thirdly, regarding the path of learning obedience: self-esteem had a significant negative impact on learning obedience (β = −0.129, *p* < 0.05), and family SES had a significant negative impact on learning obedience (β = −0.196, *p* < 0.05) in Figure 2.

(2) Mediation Effect Examination

In order to further examine the mediating role of self-esteem in the influence of family SES on learning conformity, this study employed the Bootstrap estimation method with 5000 random resamples from a sample size of 339. The study estimated the 95% confidence interval (BootCI) of the mediation effect and the proportion of the mediation effect. The results are presented in Table 4.

This table presents the results of a mediation analysis, specifically examining the indirect effects of Family Socioeconomic Status (SES) on various aspects of learning behavior through self-esteem. The analysis employs a structural equation modeling (SEM) framework to estimate the direct and indirect effects.

Predicted Path: This column outlines the hypothesized causal chain. For instance, “Family SES => Self-esteem => Learning Abidance” suggests that Family SES influences Learning Abidance through its effect on Self-esteem.

Total Effect (c): This represents the total effect of Family SES on the outcome variables (Learning Abidance, Learning Compliance, Learning Obedience) without considering the mediator (Self-esteem). The values provided indicate a statistically significant total effect of Family SES on each learning behavior.

Total Effect (a) & (b):

Indirect Effect (a*b): This column shows the indirect effect of Family SES on the learning behaviors through Self-esteem. Calculated as the product of Total Effect (a) and Total Effect (b), it quantifies the mediated effect. For example, an Indirect Effect of 0.216 for Learning Abidance suggests that Family SES positively influences Learning Abidance through its impact on Self-esteem.

95% BootCI of Indirect Effect: This column provides the 95% bootstrap confidence interval for the indirect effect. If this interval does not include zero, it indicates a statistically significant indirect effect. For instance, the confidence interval of 0.149~0.287 for Learning Abidance confirms the significance of the mediated effect.

Direct Effect (c′): This represents the direct effect of Family SES on the learning behaviors after accounting for the mediation through Self-esteem. Values like 0.429 ** for Learning Abidance indicate a significant direct effect even when controlling for the mediator.

Proportion of Effect: This indicates the proportion of the total effect that is mediated by Self-esteem. For example, a value of 0.3349 for Learning Abidance means that approximately 33.49% of the total effect of Family SES on Learning Abidance is mediated through Self-esteem.

In summary, this table outlines the mediating effects of self-esteem in the relationship between socioeconomic status and various learning behaviors, specifically learning abidance, learning compliance, and learning obedience, with proportions of 33.49%, 33.33%, and 39.77%, respectively.

## 5. Conclusions

(I) Key Research Findings

Variations in family SES, types of learning conformity, and self-esteem were observed among adolescents from different groups.

(1) The study reveals that there are no discernible gender or grade disparities in the patterns of learning conformity among adolescents. This finding contradicts previous research, which has demonstrated notable variations in educational support and learning incentives between genders, particularly in relation to the family’s SES [36]. Conventionally, it has been observed that compared to males, females often receive less economic support from their families and less emphasis is placed on their psychological learning needs. Furthermore, the self-esteem levels of female adolescents are typically lower.

Interestingly, our results suggest that when it comes to learning conformity, gender and grade do not play a significant role. This indicates that learning motivation may not vary significantly based on gender, contrary to what might be expected from previous research. This raises critical questions about the influence of other factors, such as personal interests, teaching methodologies, or school environments, which could potentially have a more significant impact on learning conformity than gender or grade alone.

Moreover, this study highlights the need for a deeper understanding of the complexities surrounding learning motivation and conformity among adolescents. It suggests that future research should explore a broader range of variables, including individual differences, psychosocial factors, and educational contexts, to gain a comprehensive understanding of what drives learning compliance in this age group. By doing so, educators and policymakers can develop more targeted and effective strategies to enhance adolescents’ engagement and success in learning.

(2) Adolescents from rural backgrounds tend to demonstrate stronger tendencies towards learning abidance and obedience than those hailing from urban environments. This study concurs with this observation, revealing that rural adolescents are more prone to adhere to learning rules and show obedience, in contrast to their urban peers. This finding raises intriguing questions about the influence of the environment on learning attitudes. It suggests that rural environments, perhaps due to their more traditional and structured nature, foster a culture of abidance and obedience in learning, whereas urban settings, with their greater diversity and exposure to different ideas, may cultivate more independent and critical thinking among adolescents. However, it is important to note that while abidance and obedience may be valued traits in certain educational settings, they should not overshadow the importance of encouraging critical thinking and autonomy in learning.

(3) Adolescents attending key high schools have significantly better family SES and self-esteem levels than those attending regular high schools. These results also indicate significant differences among adolescents from different groups in terms of their places of origin and school levels. Specifically, adolescents attending key high schools have significantly better family SES and self-esteem levels than those attending regular high schools.

(II) The influence of family SES varies on different types of learning conformity, with positive effects on learning abidance and learning compliance, and a negative effect on learning obedience.

Adolescents are at a crucial stage of self-awareness, individual development, and cognitive transformation. They exhibit considerable plasticity in subjective cognition, psychological development, and cognitive tendencies. However, when faced with inadequate family support or unfavorable educational regulation, adolescents may experience phenomena such as self-doubt and self-denial. In fact, most studies indicate that a supportive family environment, economic stability, and abundant social capital contribute to the stimulation of students’ learning potential [37]. The significance of family factors not only involves the cognitive and psychological maturity of adolescents but also relates to their academic performance and learning motivation in school. This study suggests that the influence of family SES on different types of learning conformity varies.

(1) Family SES exerts a favorable impact on adolescents’ learning abidance and compliance. According to the study, adolescents from families with elevated socioeconomic status demonstrate a stronger propensity towards motivations related to learning abidance and compliance. This finding suggests that the cumulative influence of family economics and parental education level on children’s learning psychology [38] indirectly bolsters students’ intrinsic motivation to learn, which in turn fosters a tendency towards learning abidance. Moreover, families with a higher SES cultivate a competitive mindset among students, urging them to vigorously pursue academic achievements and advancement opportunities [22], thereby promoting a disposition towards learning compliance.

(2) Family SES has a negative influence on adolescents’ learning obedience, which is often characterized as a utilitarian motivation for learning. Concurrently, the research findings indicate that adolescents from families with lower socioeconomic status are more inclined towards motivations for learning obedience. This tendency towards obedience among lower-SES adolescents may stem from their awareness of limited resources and opportunities. In such contexts, obedience becomes a means to access educational and social opportunities that are otherwise unavailable, reflecting a pragmatic approach to learning and advancement.

Moreover, this utilitarian mindset might be reinforced by societal expectations and pressures, where conforming to norms and authority figures is seen as a pathway to success. However, it’s important to note that while obedience might offer short-term gains, it might also hinder critical thinking and autonomous learning in the long run [39]. Educators and policymakers should be mindful of this trade-off and strive to create educational environments that foster both obedience and critical thinking [40], allowing adolescents from all SES backgrounds to develop a balanced approach to learning and growth.

(III) Self-esteem Mediates the Influence of Family SES on Learning Conformity.

This study also constructed a mediation model based on the “family SES → self-esteem → learning conformity” pattern. The research found that family SES has a positive predictive mechanism on self-esteem (β = 4.540 **, *p* < 0.01). The path analysis results support the following pathways:

(1) Family SES → self-esteem → learning abidance: Self-esteem plays a partially mediating role in the process of family SES influencing learning abidance. As shown in Table 3, family SES not only positively influences students’ tendencies towards learning abidance but also affects learning abidance through self-esteem. Parents with higher socioeconomic status often cultivate their children’s subjective initiative in learning, as well as higher levels of self-awareness and self-esteem, thus enhancing adolescents’ motivation for learning abidance.

(2) Family SES → self-esteem → learning compliance: Self-esteem plays a partially mediating role in the process of family SES influencing learning compliance. Family SES positively influences learning compliance, and the indirect effect of self-esteem between family socioeconomic status and learning compliance also exhibits partial mediation.

Compliance, in this context, represents a conformity behavior at the emotional level, where individuals align their actions and beliefs with external norms, expectations, or authorities. This emotional aspect of compliance goes beyond mere obedience to rules or instructions; it involves a deeper, affective commitment to social norms and expectations.

Adolescents from families with higher SES often have greater access to resources and opportunities that foster positive self-esteem. When these adolescents perceive themselves as valued and capable, they are more likely to comply with learning expectations set by their families, schools, or society. This compliance is not just a superficial act but is rooted in a sense of belonging, acceptance, and the desire to maintain positive relationships with authority figures and peers.

Moreover, adolescents with high self-esteem tend to internalize social norms and values, viewing them as integral to their identity. This internalization leads to a deeper level of compliance, where adhering to these norms becomes an extension of their self-identity, rather than an external imposition.

In summary, self-esteem partially mediates the relationship between family SES and learning compliance, particularly at the emotional level. Adolescents from higher SES families, nurtured by resources and support that bolster their self-esteem, are more likely to exhibit compliance as a form of emotional conformity to social norms and expectations.

(3) Family SES → self-esteem → learning obedience: Further analysis of the mediation effect shows that the indirect effect of self-esteem between family SES and learning obedience manifests as mediation. The direct effect of independent family SES on learning obedience is negative. This suggests that, while a higher SES might provide more resources and opportunities, it does not directly translate into a higher level of learning obedience, possibly due to a complex interplay of factors such as parenting styles, educational expectations, and the individual’s perception of their academic abilities.

Obedience, in this context, is viewed as a utilitarian form of conformity behavior. Adolescents from lower SES families may be more motivated to conform and obey authority figures, seeing it as a means to access valuable resources and opportunities that they might not otherwise have. This utilitarian approach to learning obedience could stem from a practical understanding of their societal and educational environment.

The mediating role of self-esteem becomes crucial in this dynamic. Adolescents with higher self-esteem, often nurtured in higher SES families through positive reinforcement and support, may find it easier to conform to learning expectations, not just for extrinsic rewards but also because they value their own achievements and abilities. Conversely, adolescents from lower SES backgrounds, who may lack this nurturing environment, could find obedience to be a necessary strategy for advancement, focusing more on the potential benefits it may bring.

It is important to note that while obedience can be a useful strategy, especially in structured learning environments, an overemphasis on it may hinder critical thinking and creativity. Educators and parents should strive to balance the need for obedience with encouraging independent thought and problem-solving skills. By fostering environments that value both conformity and creativity, we can help adolescents from all SES backgrounds develop a more holistic and well-rounded approach to learning.

## 6. Limitations of the Study

Generalizability: The generalizability of the findings from this study, which focused on learning conformity behaviors among high school students aged 15–18, may be constrained by the specificity of our sample. Factors like the geographical locale and cultural background of the participants could potentially impact the broader applicability of our results. Conducting the study within a particular region and with a specific demographic subset, in this case, high school students, might hinder the extrapolation of our findings to a more extensive audience.

Study Design: Our research employed a cross-sectional design, inherently limiting our capacity to definitively establish causal connections between variables. Despite the introduction of mediation models, stronger evidentiary support for causality would be garnered through longitudinal or experimental study designs.

Data Accuracy: The accuracy of our data might be affected by the study’s heavy reliance on self-reported metrics, including family SES, personal self-esteem, and learning behaviors. Respondents’ answers could be influenced by social desirability or other biases, introducing a potential measurement bias.

Particular Cultural Background:The current research was conducted within a relatively homogeneous cultural group, which may restrict the generality and applicability of the findings. To overcome this limitation, we intend to extend the analysis of conformity behavior to diverse cultural groups in future studies. This will enable us to gain a more comprehensive understanding of compliance behavior across different cultural contexts and potentially derive more universal conclusions. Through such research, we aspire to develop a deeper understanding of the impact of cultural differences on conformity behavior and make meaningful contributions to the fields of cross-cultural psychology and sociology.

## 7. Research Implications

Importance of Family SES in Adolescent Learning Behavior: This study emphasizes the influence of family SES on adolescent learning abidance, obedience, and compliance. It highlights the critical role of the family environment in shaping adolescent learning behaviors and attitudes, underscoring the need to address family factors in understanding adolescent learning.

Mediating Role of Self-esteem in the Learning Process: The study finds that self-esteem mediates the relationship between family SES and learning behavior. This suggests the importance of considering adolescent self-esteem levels and how enhancing self-esteem can promote positive learning behaviors.

Consideration of Regional and Cultural Differences: The findings of this study may be more representative of specific regions or cultural backgrounds. Therefore, it is important to consider regional and cultural differences in both research and practice to ensure the applicability and effectiveness of the results.

Significance of Family Educational Support: Higher family SES is associated with greater educational support, including financial support, social capital, and cultural capital. Hence, educational policies and practices should focus on providing broader and more effective family educational support to facilitate adolescent learning development.

Recognition of Individual Differences: Despite exploring the influence of family factors on learning behavior, individual differences persist. Therefore, when designing educational interventions, it is essential to consider individual differences and implement targeted measures to meet the needs and potentials of different adolescents.

## Figures and Tables

**Table 1:** Reliability and Convergent Validity of the Learning Conformity Scale.

Factor Items	Cronbach’s Alpha	Average Variance Extracted (AVE)	Composite Reliability (CR)
Learning Abidance	0.965	0.847	0.965
Learning Compliance	0.973	0.879	0.973
Learning Obedience	0.936	0.786	0.936

**Table 2:** Discriminant Validity of the Learning Conformity Scale.

Factor Items	Learning Abidance	Learning Compliance	Learning Obedience
Learning Abidance	0.909		
Learning Compliance	0.433	0.926	
Learning Obedience	0.217	0.311	0.887

**Table 3:** Mean, Standard Deviation, and Pearson Correlation Analysis of Variables (N = 339).

Variable	Mean	Standard Deviation	1	2	3	4	5	6	7	8	9	10
Gender (1)	1.608	0.489	1									
Birthplace (2)	1.487	0.501	−0.064	1								
Grade (3)	2.578	1.11	0.059	-0.093	1							
School Level (4)	1.369	0.483	0.101	0.308 **	0.043	1						
Subject Category (5)	2.094	0.816	0.119	0.025	-0.025	-0.036	1					
Family SES (6)	12.241	5.039	−0.520 **	−0.568 **	0.036	−0.341 **	0.376 *	1				
Self-esteem (7)	30.1	11.731	−0.530 **	−0.566 **	0.025	−0.676 **	0.485 **	0.523 **	1			
Learning Abidance (8)	11.139	7.434	−0.128	−0.544 **	0.015	−0.529 **	0.034	0.627 **	0.148 *	1		
Learning Compliance (9)	13.263	8.243	−0.126	0.182	−0.014	0.002	0.368 *	0.412 **	0.425 **	−0.421 **	1	
Learning Obedience (10)	7.67	5.02	0.182	−0.706 **	0.027	−0.594 **	−0.061	−0.638 **	−0.499 **	−0.638 **	−0.328 **	1

Note: Gender is represented by a dummy variable, where 1 denotes male and 2 represents female. Birthplace is indicated by a dummy variable, with 1 signifying urban and 2 signifying rural. School Level is designated by a dummy variable, where 1 corresponds to a key high school and 2 to a regular high school. Subject Category is expressed through a dummy variable, with 1 for humanities, 2 for science, and 3 for arts. * *p* < 0.05 ** *p* < 0.01

**Table 4:** Analysis of Bootstrap Significance Test Results for Mediation Effects.

Predicted Path	c	Total Effect (a)	Total Effect (b)	Indirect Effect (a*b)	95% BootCI of Indirect Effect	Direct Effect (c’)	Test Conclusion	Proportion of Effect
Family SES => Self-esteem => Learning Abidance	0.645 **	4.540 **	0.048 **	0.216	0.149~0.287	0.429 **	Partial Mediation	0.3349
Family SES => Self-esteem => Learning Compliance	0.498 **	4.540 **	0.037 **	0.166	0.112~0.225	0.332 **	Partial Mediation	0.3333
Family SES => Self-esteem => Learning Obedience	−0.533 **	4.540 **	−0.047 **	−0.212	−0.305~−0.134	−0.321 **	Partial Mediation	0.3977

* *p* < 0.05 ** *p* < 0.01.

## Data Availability

The raw data supporting the conclusions of this article will be made available by the authors, without undue reservation.
